# Office Hysteroscopy as a Valid Tool for Diagnosis of Genital Tract Lesions in Females with Intact Hymen

**DOI:** 10.1155/2019/4074975

**Published:** 2019-11-11

**Authors:** Hui-Yu Huang, Yi-Ting Huang, Kai-Yun Wu, Yu-Ying Su, Cindy Hsuan Weng, Chin-Jung Wang

**Affiliations:** ^1^Department of Obstetrics and Gynecology, Chang Gung Memorial Hospital at Taipei, Taipei, Taiwan; ^2^Department of Obstetrics and Gynecology, Chang Gung Memorial Hospital at Linkou, Kweishan, Taoyuan, Taiwan; ^3^Chang Gung University College of Medicine, Taoyuan, Taiwan

## Abstract

**Background:**

To evaluate the feasibility and applicability of using office hysteroscopy in women with intact hymen.

**Methods:**

We recruited 836 patients with intact hymen with different indications who underwent diagnostic hysteroscopy without anesthesia in an outpatient setting from 2007 to 2016 at Chang Gung Memorial Hospital at Linkou.

**Results:**

Patients' mean age was 35 ± 10.6 years (range 3–69 years). Most patients (86.4%) with postmenopausal bleeding had intrauterine lesions, and they were especially at high risk (50%) for endometrial hyperplasia or malignancy. Five hundred thirty (63.3%) patients had histologic findings confirming concordance between hysteroscopic and histologic findings. Submucosal myoma had the highest concordance (96.3%), whereas endometrial hyperplasia had the lowest concordance (50%). Forty-eight patients (5.7%) had endometrial hyperplasia, and 35 patients (4.2%) had endometrial malignancy. Two patients who were thought to have nonspecific endometrial thickening actually had endometrial pathology.

**Conclusions:**

Hysteroscopy through vaginoscopic approach is feasible and well-tolerated in the patients with intact hymen. This outpatient procedure provides accurate evaluation of lesions of the genital tract and should be considered in patients without a history of intercourse.

## 1. Introduction

Abnormal uterine bleeding (AUB) is a common symptom, accounting for one-third of outpatient visits to gynecologists among premenopausal women [1]. Pediatric and adolescent patients with gynecological diseases also commonly present with vaginal discharge and bleeding. Obtaining a detailed patient history and pelvic examination is crucial for determining the etiology of AUB. However, a vaginal examination is difficult to perform in women with an intact hymen. Further imaging studies may be indicated, and transabdominal ultrasonography has been the modality of choice in adolescent gynecology or for virginal patients without a history of intercourse. Nevertheless, optimal evaluation of the endometrium generally requires transvaginal ultrasonography or hysterosonography, which enables physicians to detect intrauterine abnormalities. Owing to these limitations, prepubertal or virginal patients may have delayed diagnosis and improper treatment, especially for some serious diseases, such as endometrial hyperplasia and malignancies.

Hysteroscopy is a valuable diagnostic tool for evaluating intrauterine lesions and anomalies. Inserting a speculum and applying a tenaculum to the cervix may facilitate the procedure in traditional hysteroscopy [2]. However, the use of these instruments may damage the vagina or hymen and cause pain; thus, analgesics or anesthesia may be required to reduce patient discomfort. Bettocchi et al. proposed a vaginoscopic approach in 1997 for diagnostic hysteroscopy [3]. This nontraumatic method was performed by inserting the hysteroscope into the vagina, further advancing it into the uterine cavity through the cervical canal. With distention fluid, the vaginal walls, the fornices, and the cervix were well visualized. Various studies have demonstrated that the vaginoscopic technique causes less pain and has advantages for women with a stenotic or narrow vagina [3ߝ^5^].

At our hospital, outpatient hysteroscopy was introduced in 1993, and it became a routine office procedure 1 year later. We have used flexible hysterofibroscopy for more than 15 years and rigid hysterofibroscopy since July 2013. In this retrospective study, we evaluated the results of patients with an intact hymen who underwent diagnostic hysteroscopy without anesthesia, and we aimed to assess the feasibility and applicability of hysteroscopy in this population.

## 2. Materials and Methods

Institutional review board approval was obtained for the study. From January 2007 to December 2016; 836 patients with an intact hymen underwent diagnostic hysteroscopy in an outpatient setting at our center. Indications were persistent vaginal discharge, abnormal uterine bleeding, suspected Müllerian anomalies, and suspicious findings on an ultrasound examination.

Informed consent was obtained from all patients before the procedure. They were informed that they may experience menstrual-type cramps during or after the examination. They were assured that the hysteroscopy could be discontinued at any time upon their request. All procedures were performed without the use of premedication, analgesics, or anesthesia. Juvenile patients were examined by the company of their family members. Eleven patients with anatomic impediments (e.g., an acutely flexed uterus) were managed with the aid of a tenaculum. A 3.1-mm diameter flexible hysterofiberscope (HYF-XP; Olympus Corp., Shinjuku-ku, Tokyo, Japan) or a rigid single-flow hysteroscope (Karl Storz®, Tuttlingen, Germany) with a 2.7-mm diameter and an external sheath of 3.2 mm was used, depending on the equipment that was readily available.

A 0.9% normal saline solution was used as the distending medium, propelled by an electronic pump, with an intrauterine pressure maintained at 45 mmHg. The procedure was performed by an experienced hysteroscopist (CJW). After inserting the hysteroscope, the cervix, cervical canal, fornices, vaginal walls, tubal ostium, uterine cavity, and endometrium were carefully inspected (video clip). Illumination was provided by a high-intensity cold light source supplied by a fiber-optic lead. Images were viewed on a high-resolution color television monitor, and patients were encouraged to observe images of their procedure on the screen.

## 3. Results

Hysteroscopy was finished within 10 minutes in all patients. Eight patients (0.95%) had vagal reactions during the examination and recovered fully after bed rest and hydration. No complications required hospitalization or led to further morbidity. The integrity of the hymen was preserved in all patients. We encountered anatomic impediments in 11 of 836 patients (1.3%), and these patients were managed successfully with the use of a tenaculum.

Characteristics of the 836 patients (age range, 3–69 years; mean, 35.0 ± 10.6 years) are shown in Table 1. Most patients (748 out of 836, 89.5%) were premenopausal, 66 (7.9%) were children or adolescents, and the remaining 22 (2.6%) were postmenopausal. The most common indication for hysteroscopy was intermenstrual bleeding, followed by suspicious intrauterine outgrowths on a transabdominal ultrasonogram. Three hundred twenty-five patients had more than one indication.

The relationships between indications and hysteroscopic findings are shown in Table 2. More than half of the patients who presented with persistent vaginal discharge (9/14, 64.3%) or vaginal bleeding (11/21, 52.4%) had normal hysteroscopic findings. Two patients who had persistent vaginal discharge had a foreign body in their vagina (doll hair in 1 patient and a pen cap in the other patient). Women who had heavy menstrual bleeding (93/98, 94.9%) or intermenstrual bleeding (446/681, 65.5%) also had intrauterine lesions observed by hysteroscopy. Submucosal myoma constituted the main lesion causing heavy menstrual bleeding, whereas an endometrial polyp accounted for the main lesion leading to intermenstrual bleeding. Although postmenopausal patients were the minor group (22 out of 836, 2.6%) in this study, most patients (19 out of 22, 86.4%) with postmenopausal bleeding (PMB) had intrauterine lesions, and they were at especially high risk (50.0%) of endometrial hyperplasia or malignancy. Patients clinically suspected to have Müllerian duct anomalies were all confirmed by hysteroscopy. Women who were suspected to have intrauterine outgrowths mostly (82.0%) had positive hysteroscopic findings, mainly an endometrial polyp.

Five hundred and thirty (63.4%) patients had histologic diagnosis and the concordance between hysteroscopic and histologic findings was 87.2% (Table 3). Submucosal myoma had the highest concordance (96.3%), whereas endometrial hyperplasia had the lowest concordance (50.0%). Overall, 48 out of 836 patients (5.7%) were found to have endometrial hyperplasia, and 35 patients (4.2%) had endometrial malignancy. Two patients who were thought to have nonspecific endometrial thickening actually had endometrial hyperplasia and endometrial malignancy.

Of 836 patients, 556 patients (66.5%) underwent transabdominal ultrasonography and hysteroscopy concomitantly, and 116 of these patients (20.9%) reportedly had normal ultrasonograms (Table 4). However, 67 patients (57.8%) who had negative ultrasonogram findings showed endometrial lesions on hysteroscopy.

## 4. Discussion

Nulliparity, late menopause, obesity, Diabetes mellitus, unopposed estrogen therapy, and polycystic ovarian syndrome were risks factors for developing endometrial malignancy. Nulliparous women have two to three times the risk of parous women [6]. In this study, we observed that virginal women with PMB had 86.4% of having intrauterine lesions. Ten of the 22 (45.5%) postmenopausal women were found to have endometrial malignancy. Hysteroscopy is suggested to be performed in the case of AUB in postmenopausal women [7, 8]. A recent multicenter randomized controlled trial also suggested that hysteroscopy is warranted to detect focal (pre)malignancies in women with PMB [9]. Furthermore, hysteroscopy allowed directed biopsies of suspicious lesions, improving the accuracy of diagnosis compared with curettage [10]. Therefore, hysteroscopy is an important diagnostic tool to evaluate patients with risk factors for endometrial hyperplasia and malignancy, and those who do not respond to medical treatment for AUB [11, 12].

Numerous papers have reported that the accuracy of hysteroscopy for diagnosing endometrial malignancy is high, but for endometrial hyperplasia it is only moderate [13–15]. Our results agree with these findings. Performing endometrial biopsy when hysteroscopy shows unevenly shaped and thick endometrial mucosa can reduce the incidence of misdiagnosis.

Our study showed that 50.8% of patients who had unremarkable intrauterine lesions revealed by transabdominal sonography actually had intrauterine lesions. Studies reported that transvaginal ultrasonography is superior to transabdominal ultrasonography for diagnosing intrauterine lesions [16, 17]. Virginal women cannot undertake transvaginal ultrasound examination. Physicians should consider hysteroscopy as a complementary tool to evaluate intracavitary lesions in women with an intact hymen, especially for those who are refractory to medical management [18, 19].

The strengths of this study were that all examinations were performed in an office setting without anesthesia or analgesia, demonstrating its good tolerance and feasibility, even in children. Further, only a skillful endoscopist (CJW) performed the examinations, making image interpretation consistent. This also explains why only 11 (1.3%) patients needed additional assistance (tenaculum) to overcome anatomic obstacles. For a markedly anteverted and retroverted uterus, a tenaculum was applied to the posterior lip and anterior lip of the cervix, respectively, under hysteroscopic guidance. By doing so, the axis of the uterus became more horizontal, facilitating passage of the hysteroscope through the internal cervical os. Only a single examiner is also one of the limitations of this study. That is, the results may not be suitable for entire gynecologic public. Besides, we did not assess the pain scores, and the diagnoses were not histologically confirmed in all patient.

Our study suggests that hysteroscopy is feasible and well tolerated in virginal patients of all age. There was no need for anesthesia or analgesics for the procedure. Physicians should consider hysteroscopy through the vaginoscopic approach to explore the genital tract and to evaluate intrauterine lesions in this population, particularly for postmenopausal women.

## Figures and Tables

**Table 1:** Patients' characteristics.

	Value
Age (years)	35.0 ± 10.6 (3–69)
*Genital status*	
Premenarche (<9 years)	9 (1.1%)
Preteen (9–12 years)	8 (1.0%)
Teen (13–18 years)	49 (5.9%)
Premenopausal (>18 years)	748 (89.5%)
Postmenopausal	22 (2.6%)

*Indications for hysteroscopy * ^∗^	
Persistent vaginal discharge	14
Vaginal bleeding	21
Heavy menstrual bleeding	98
Intermenstrual bleeding	681
Postmenopausal bleeding	22
Suspected mullerian anomalies	12
Suspected intrauterine outgrowth	556

Values are presented as a mean ± standard deviation (range) or number (%). ^∗^Some patients had more than one indication.

**Table 2:** Correlation between indications and hysteroscopic findings.

Indication	Hysteroscopy							
	No.	Normal	Foreign body	Vaginal or uterine septum	Em polyp	Submucosal myoma	Em hyperplasia or malignancy	Miscellaneous
Persistent vaginal discharge	14	9 (64.3)	2^a^ (14.3)	1 (7.1)	0	0	0	2^b^ (14.3)
Vaginal bleeding	21	11 (52.4)	0	1 (4.8)	3 (14.3)	1 (4.8)	0	5^c^ (23.8)
Heavy menstrual bleeding	98	5 (5.1)	0	0	29 (29.6)	42 (42.9)	22 (22.4)	0
Intermenstrual bleeding	681	235 (34.5)	0	9 (1.3)	298 (43.8)	34 (5.0)	85 (12.5)	19^d^ (2.8)
Postmenopausal bleeding	22	3 (13.6)	0	0	6 (27.3)	2 (9.1)	11 (50.0)	0
Suspected mullerian anomalies	12	0	0	12 (100)	0	0	0	0
Suspected intrauterine outgrowth	556	85 (15.3)	0	0	283 (50.9)	71 (12.8)	102 (18.3)	15^e^ (2.7)

No. = number; Em = endometrial. Values are presented as *n* (%). ^a^Doll hairs in 1 child, and pen caps in another child. ^b^Vaginitis. ^c^A urethral polyp in 1 patient, and vaginal cuff granulation tissue (after hysterectomy) in 4 patients. ^d^A vaginal stone in 1 patient, non-specific endometrial thickening in 18 patients. ^e^Nonspecific endometrial thickening.

**Table 3:** Correlations between hysteroscopic and histologic findings in 530 patients.

Hysteroscopic findings	Histologic findings						
	No.	Normal	Endometrial polyp	Submucosal myoma	Endometrial hyperplasia	Endometrial malignancy	Concordance (%)
Endometrial polyp	333	3 (0.9)	319 (95.8)	5 (1.5)	3 (0.9)	3 (0.9)	95.8
Submucosal myoma	80	0	1 (1.3)	77 (96.3)	0	2 (2.5)	96.3
Nonspecific endometrial thickening	13	9 (69.2)	2 (15.4)	0	1 (7.7)	1 (7.7)	69.2
Endometrial hyperplasia	80	15 (18.8)	13 (16.3)	0	40 (50.0)	12 (15.0)	50
Endometrial malignancy	24	3 (12.5)	0	0	4 (16.7)	17 (70.8)	70.8

No. = number. Values are presented as *n* (%).

**Table 4:** Correlations between normal transabdominal ultrasound report and hysteroscopic findings in 116 patients.

Transabdominal ultrasound	Hysteroscopic findings					
	No.	Normal	Endometrial polyp	Submucosal myoma	Endometrial hyperplasia	Endometrial malignancy
Normal	116	49 (42.2)	35 (30.2)	23 (19.8)	8 (6.9)	1 (0.9)

No. = number. Values are presented as *n* (%).

## Data Availability

The data used to support the findings of this study are available from the corresponding author upon request.

## Supplementary Materials

Supplementary Materials The description of the video was “Flexible Office Hysteroscopy with the Vaginoscopic approach”.
